# Developing a Highly Stable PLGA-mPEG Nanoparticle Loaded with Cisplatin for Chemotherapy of Ovarian Cancer

**DOI:** 10.1371/journal.pone.0025433

**Published:** 2011-09-26

**Authors:** Lihua Cheng, Chengmeng Jin, Wen Lv, Qiuping Ding, Xu Han

**Affiliations:** 1 Zhejiang-California International NanoSystems Institute, Zhejiang University, Zhejiang, People's Republic of China; 2 The Second Affiliated Hospital Zhejiang University College of Medicine, Zhejiang University, Zhejiang, People's Republic of China; National Institutes of Health, United States of America

## Abstract

**Background:**

Cisplatin is a potent anticancer drug, but its clinical application has been limited due to its undesirable physicochemical characteristics and severe side effects. Better drug formulations for cisplatin are highly desired.

**Methodology/Principal Findings:**

Herein, we have developed a nanoparticle formulation for cisplatin with high encapsulation efficiency and reduced toxicity by using cisplatin-crosslinked carboxymethyl cellulose (CMC) core nanoparticles made from poly(lactide-co-glycolide)-monomethoxy-poly(polyethylene glycol) copolymers (PLGA-mPEG). The nanoparticles have an average diameter of approximately 80 nm measured by transmission electron microscope (TEM). The encapsulation efficiency of cisplatin in the nanoparticles is up to 72%. Meanwhile, we have also observed a controlled release of cisplatin in a sustained manner and dose-dependent treatment efficacy of cisplatin-loaded nanoparticles against IGROV1-CP cells. Moreover, the median lethal dose (LD_50_) of the cisplatin-loaded nanoparticles was more than 100 mg/kg by intravenous administration, which was much higher than that of free cisplatin.

**Conclusion:**

This developed cisplatin-loaded nanoparticle is a promising formulation for the delivery of cisplatin, which will be an effective therapeutic regimen of ovarian cancer without severe side effects and cumulative toxicity.

## Introduction

Cisplatin-based therapeutic regimens have been established as the standard first-line chemotherapy for patients with ovarian cancer since the mid-1980s [1]–[3]. However, its application has been limited because of severe side effects and cumulative toxicity on major organs such as liver, kidney, heart and nervous system by intravenous administration [4]–[7]. In recent years, numerous nano-sized drug carriers have been developed to minimize side effects of cisplatin and enhance its antitumor efficacy through the forms of micelles such as poly(aspartic acid)-poly(ethylene glycol) micelles, liposomes such as pegylated liposomes and solid lipid nanoparticles during cancer therapy [8]–[13]. Soluble drug-polymer conjugates have also been developed to increase the solubility of cisplatin. Such conjugates include cisplatin complexes with polycarboxylates, poly(amidoamines), polyamidoamine dendrimers and polyacrylic chains. These systems can reduce toxicity and achieve ideal efficacy of chemotherapy [14]–[16].

Poly(lactic-co-glycolic) acid (PLGA) microparticles have also been used for cisplatin entrapment due to its biocompatible and biodegradable properties [17], [18]. Avgoustakis K et al. have demonstrated that the intravenous administration of PLGA-mPEG nanoparticles loaded with cisplatin results in a significant prolongation of cisplatin presence in blood of mice [19]. However, the encapsulation efficiency of cisplatin is still poor during the preparation of PLGA-mPEG nanoparticles loaded with cisplatin. Gryparis EC et al. [20] have found that nanoparticle application has the possibility to maintain adequate or more concentrations of cisplatin for 2 weeks, but relatively low PEG content of PLGA-mPEG can induce the increase of nanoparticle size. Furthermore, it is desirable to achieve a sustainable release and sufficient concentration of cisplatin in clinical settings because continuous low-dose administration of cisplatin is more effective in inducing apoptosis than a single high-dose exposure of cisplatin [21], [22].

The main objective of the present work is to develop an innovative nanoparticle with a high encapsulation efficiency and sustainable release of cisplatin. In this nanoparticle delivery system, d-alpha tocopheryl polyethylene glycol 1000 succinate (TPGS) as a new efficient emulsifier beneficial for human health has been used and the biodegradable PLGA-mPEG nanoparticles with CMC cores have been developed. Meanwhile, the loading and encapsulation efficiency of cisplatin have also been determined. Moreover, the antitumor efficacy of nanoparticles loaded with cisplatin on mouse ovarian cancer model has been evaluated. In conclusion, the improved cisplatin delivery system provides a rational design for potential therapeutic application of cisplatin in ovarian cancer.

## Results

### Rational design of CMC and TPGS to prepare PLGA-mPEG nanoparticles with cisplatin

**Figure 1:**
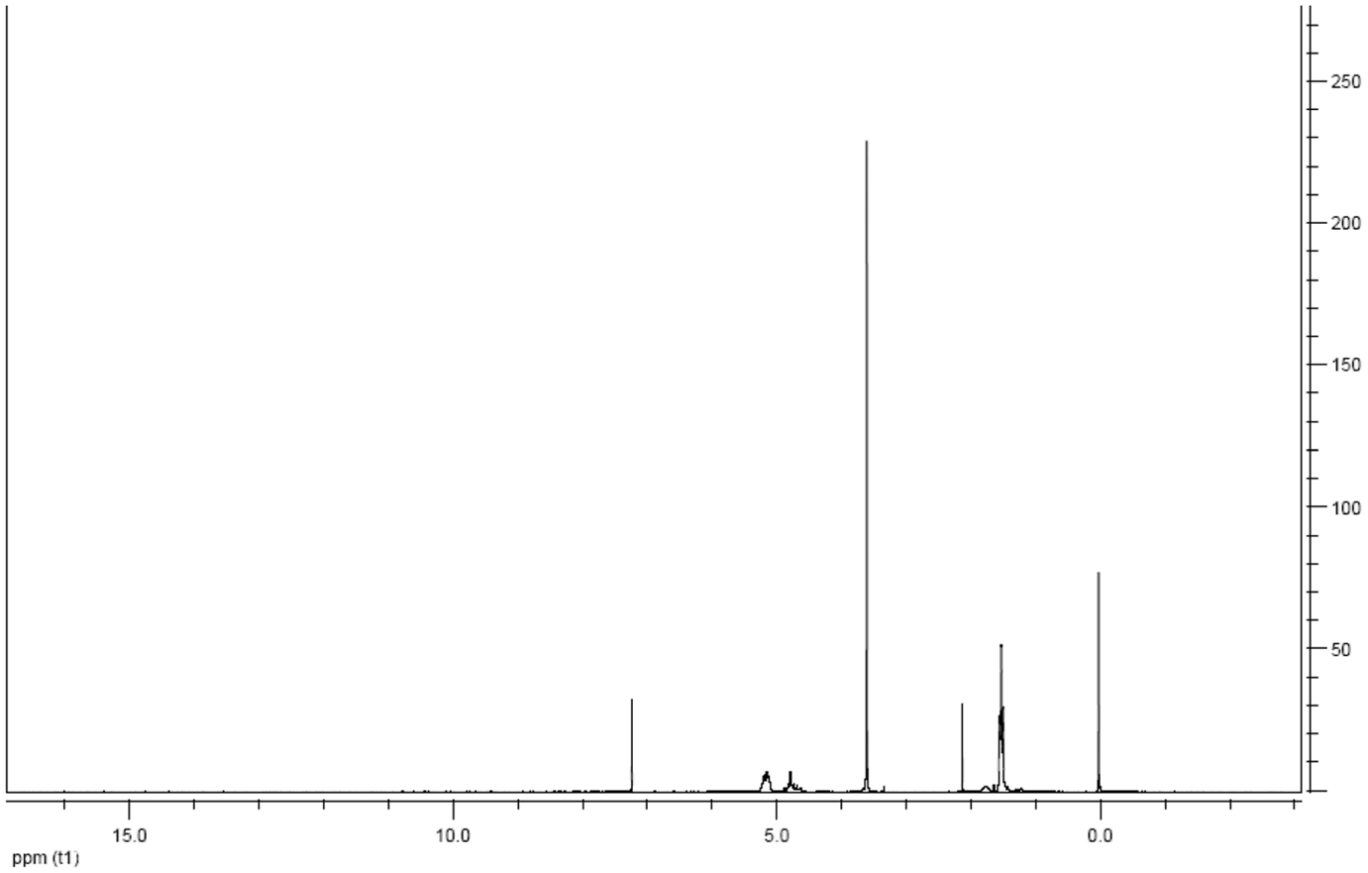
^1^H NMR spectrum of PLGA-mPEG copolymer. The peaks at 1.65 and 5.10 ppm belonged to the protons from methine (-CH) and methyl (-CH_3_) groups of PLGA segments, the peaks of methene protons (-CH_2_-) in the PEG segments located at 3.65 ppm, and the peaks of methene protons (-COCH_2_O-) located at 4.78 ppm represented conjugated segments of PLGA and mPEG.

**Figure 2:**
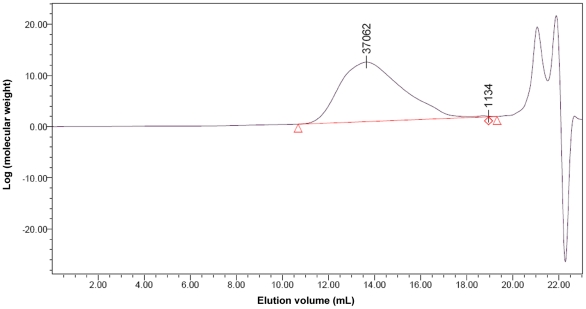
The measurement of PLGA-mPEG copolymer molecular weight by GPC. The peak represented the average molecular weight of synthesized copolymers was calculated to be 37062.

It is a challenge to prepare nanoparticles loaded with cisplatin by using PLGA-mPEG copolymers due to their physicochemical properties and preparation conditions. In this study, we conducted a rational design of nanoparticles containing cisplatin by tailoring the compositions, size and shape. Firstly, PLGA-mPEG copolymers were synthesized from lactide and glycolide in the presence of mPEG_5000_, whose hydroxyl group could initiate the ring-opening polymerization of lactide and glycolide. The structure of the synthesized copolymer was detected by ^1^H NMR in CDCl_3_, as shown in Figure 1. The peaks at 1.65 and 5.10 ppm belonged to the protons from methine (-CH) and methyl (-CH_3_) groups of PLGA segments, while the peaks of methene protons (-CH_2_-) in the PEG segments located at 3.65 ppm, and the peaks of methene protons (-COCH_2_O-) located at 4.78 ppm were the representation of conjugated segments of PLGA and mPEG so that no other peaks were detected. The only one peak in GPC spectrum (Figure 2) revealed the successful synthesis of PLGA-mPEG copolymers with high purity. The polydispersity index (PI) as a measurement of the distribution width for molecular weights was 1.78. On the basis of gel permeation chromatography (GPC) and the ratio of ^1^H peak areas between 5.10 and 3.65 ppm, the average molecular weight of synthesized copolymers was calculated to be 37062, as shown in Figure 2. The weight percentage of mPEG was 13.5%.

Secondly, a modified solvent evaporation method based on water-organic phase-water (W/O/W) multiple emulsions including a relatively simple preparation process was used to improve the encapsulation efficiency of cisplatin in nanoparticles. The observed characteristics of nanoparticles may vary largely depending on CMC concentration, TPGS concentration and the variable processes including the switch of solvent addition order. In order to achieve the optimal CMC concentration for high encapsulation efficiency, different addition amounts of CMC in the internal aqueous phase of the double emulsion system were explored. The effective encapsulation of cisplatin in nanoparticles was higher than no amount of CMC, which revealed the loading rate and encapsulation efficiency of cisplatin were 3.88 and 70.9% (w/w), respectively, as shown in Table 1. Totally 1.7 mg CMC combined with 4 mg cisplatin were selected as the optimal internal aqueous phase and the optimal concentration for TPGS was screened to be 0.035% (w/v).

**Table 1:** Entrapment efficiency and drug loading of NPs-cp (n = 3).

Batch	Entrapment efficiency	Drug loading
NPs-cp	70.9±2.6%	3.9±0.3%

The entrapment efficiency was calculated as the amount of cisplatin recovered from nanoparticles relative to the initial amount of cisplatin used for preparation. The loading efficiency of cisplatin was the amount of cisplatin recovered from nanoparticles relative to the amount of CMC and PLGA-mPEG.

**Figure 3:**
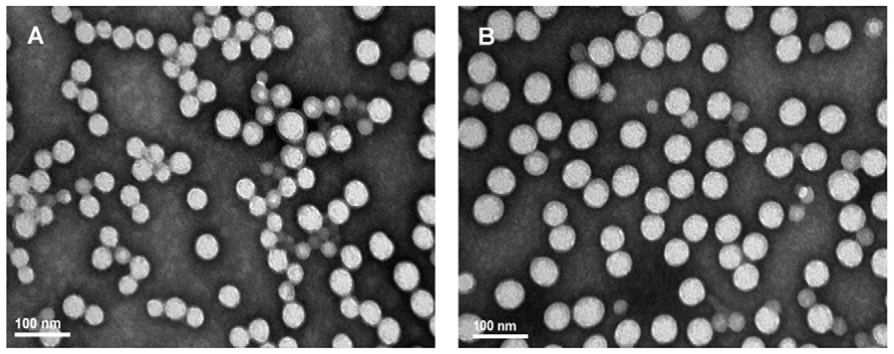
Transmission electron microscopic examination of nanoparticles. (A) blank nanoparticles; (B) cisplatin-loaded nanoparticles. Samples were stained with 1% phosphotungstic acid (50000×).

As shown in Table 2, the average size of prepared nanoparticles loaded with cisplatin ranged from 180 to 210 nm, and the loading rate of cisplatin was 3.5–4.2% (w/w). The PI was determined to be approximately 0.3, which exhibited a homogeneous distribution in diameter. CMC and PLGA-mPEG polymers conferred a negative ζ-potential with an average value of −10.1 and −9.5 mV in blank nanoparticles and cisplatin-loaded nanoparticles, respectively. No significant difference was observed in size and ζ-potential of the nanoparticles with or without cisplatin. Figure 3 showed the representative images of the external structure of nanoparticles examined by TEM. Most nanoparticles were spherical shape with smaller diameters than the diameters determined by dynamic light scattering. The morphology of nanoparticles with or without cisplatin revealed indistinguishable or similar structures.

**Table 2:** Particle size and zeta potential of blank NPs and NPs-cp (n = 3).

Batch	Particle size (nm)	Size polydispersity index (PI)	Zeta potential (mV)
Blank NPs	187±7	0.23	−10.1±0.3
NPs-cp	192±12	0.26	−9.5±0.4

NPs are nanoparticles; NPs-cp is cisplatin-encapsulated nanoparticles.

**Figure 4:**
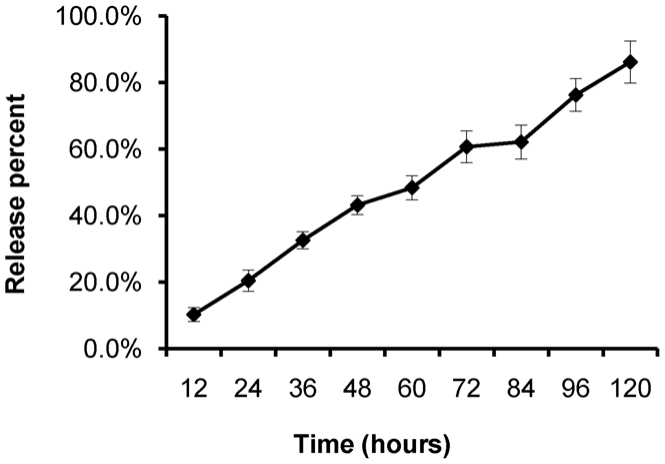
Release profiles of cisplatin-loaded nanoparticles. Cisplatin released from nanoparticles as a function of incubation time in 100 mmol/L sodium chloride solution at 37°C.

Lastly, the cumulative release profile of cisplatin from nanoparticles was measured by high performance liquid chromatography (HPLC), as shown in Figure 4. This assay method was sensitive enough to measure cisplatin in a wide concentration range with a detection limit of 0.31 ng/mL. A calibration curve was established in each experiment. The release kinetics of cisplatin from nanoparticles revealed a constant controlled release rate as the function of time. However, its stable release could be affected by CMC-cisplatin conjugation and biodegradable PLGA-mPEG copolymers. Similarly, as shown in Figure 4, the entrapment of cisplatin in nanoparticles could significantly retard the release rate of cisplatin and consequently lead to a fairly steady release over time.

### Acute toxicity of CMC core nanoparticles

**Figure 5:**
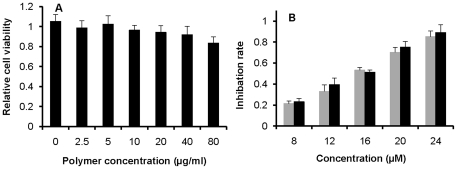
Relative cell viability of IGROV1-CP cells with different treatments. (A) Relative cell viability of IGROV1-CP cells exposed to blank nanoparticles for 72 hours with various doses. (B) Cell growth inhibition after exposure to free cisplatin (gray histogram) and cisplatin-loaded nanoparticles (black histogram) for 72 hours. The data represented as Mean ± SD.

In order to evaluate the cytotoxicity of nanoparticles without loading of drugs, the cytotoxicity of nanoparticles at the doses of 0.005, 0.02, 0.04 and 0.08 mg/mL were tested by using 96-well microplates seeded with cells at a density of 3000 cells/well. The cell growth curves of the control and nanoparticle-treated groups were similar, suggesting that the nanoparticles at the tested concentrations did not exhibit obvious toxicity on cells, as shown in Figure 5(A). In addition, the maximum amount of nanoparticles (0.08 mg/mL) and the density of 3000 cells/well were also the optimal conditions for evaluating the effect of cisplatin-loaded nanoparticles on cell growth *in vitro*.

The anticancer activity of free cisplatin and cisplatin-loaded nanoparticles on IGROV1-CP cells *in vitro* was determined. As shown in Figure 5(B), no significant difference in cytotoxicity between cisplatin-loaded nanoparticles and free cisplatin at the tested concentrations was observed (*p*>0.05). The cytotoxicty of both free cisplatin and cisplatin-loaded nanoparticles was dose-dependent. Therefore, PLGA-mPEG nanoparticles can contribute to controlled release rate of cisplatin while preserving the anticancer effectiveness of cisplatin.

In order to assess for potential toxicity risks, ICR mice were intravenously injected with cisplatin-loaded nanoparticles at various doses and the change in survival rate of the mice was examined. In all tested dose levels, death rates between groups revealed a significant difference during a 2-week waiting period (*p*<0.05). The mortality of cisplatin-loaded nanoparticles at the dose of 100 mg/kg (on the basis of cisplatin) was 40.0%, but the death rate in the 10 mg/kg cisplatin treatment group reached up to 62.5%. No health deterioration was observed in mice treated with blank nanoparticles during the observation period, and the overall behavior of the mice treated with cisplatin-loaded nanoparticles or blank nanoparticles did not exhibit an obvious difference. The LD_50_ was 8.6 mg/kg for free cisplatin and 103.4 mg/kg for cisplatin-loaded nanoparticles, as shown in Table 3. Therefore, compared with free cisplatin, the nanoparticles loaded with cisplatin can provide more safety in clinical application.

**Table 3:** LD_50_ of cisplatin and NPs-cp.

Tested nanoparticles	LD_50_ (mg/kg)		
	Male	Female	Male+Female
NPs-cp	101.3	105.6	103.4
cisplatin	8.2	8.6	8.5

Each group included ten mice; LD_50_ represented as median lethal dose.

### Safety profile of CMC core nanoparticles in IGROV1-CP xenografted nude mice

**Figure 6:**
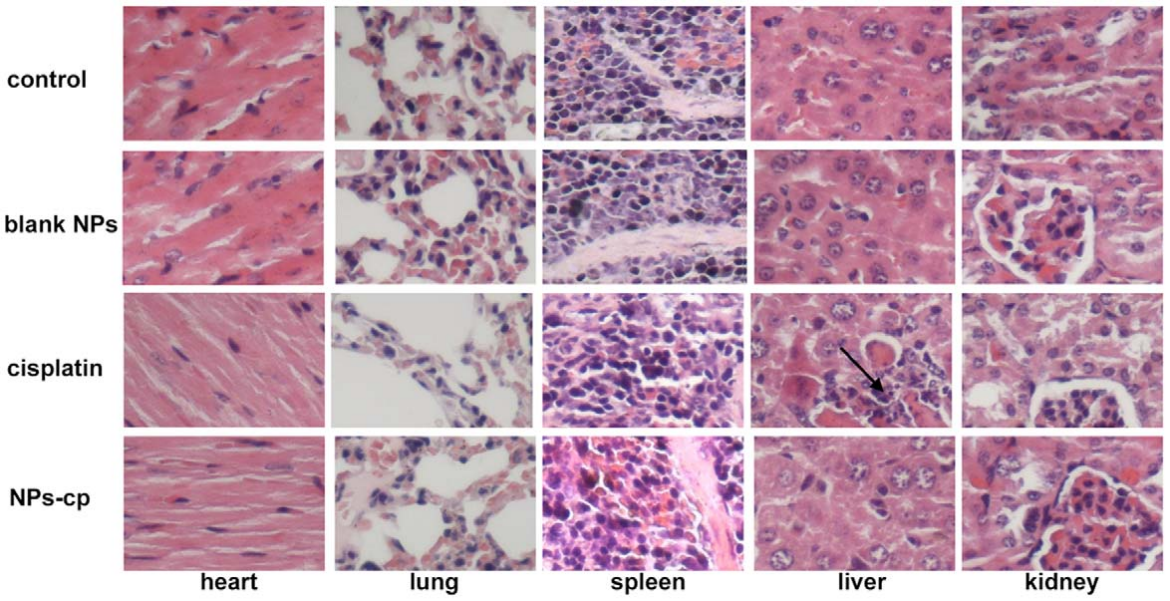
Microscopic examination of organs with HE staining. Nude athymic mice bearing IGROV1-CP xenografts were treated with saline (control), blank nanoparticles (blank NPs), cisplatin and cisplatin loaded nanoparticles (NPs-cp) at five doses every 4 days. The organs including heart, lung, spleen, liver and kidney from the mice treated with saline were shown in the first row. The organs from the mice treated with blank nanoparticles were shown in the second row. The organs from the mice treated with free cisplatin were shown in the third row. The organs from the mice treated with cisplatin-loaded nanoparticles were shown in the fourth row.

In order to observe the delayed organ toxicity on the mice within a certain treatment period, major organs including heart, liver, lung, kidney and spleen from BALB/c nude mice administered with free cisplatin and cisplatin-loaded nanoparticles were sectioned and stained with HE. No obvious histopathological change was observed in heart, lung and spleen of cisplatin-loaded nanoparticles treated mice, as shown in Figure 6. However, hepatic necrosis and pathological atrophy of kidney in mice treated with free cisplatin were observed. Similarly, the administration of cisplatin-loaded nanoparticles up to 3 mg/kg for 5 consecutive dosages did not cause a significant pathological difference in liver and kidney.

**Figure 7:**
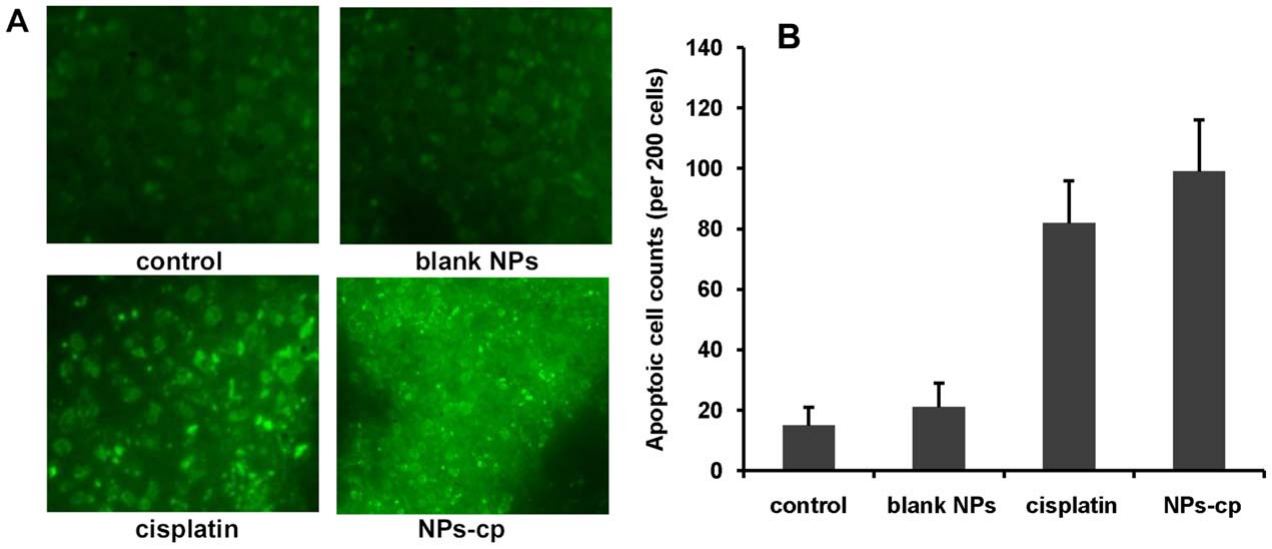
Apoptosis in tumor examined by TUNEL staining. (A) Microscopic evaluation of tumor apoptosis by TUNEL staining. Athymic mice bearing IGROV1-CP xenografts were treated with saline (control), blank nanoparticles (blank NPs), cisplatin and cisplatin loaded nanoparticles (NPs-cp) at five doses. (B) The average apoptotic cell counts were calculated on the basis of TUNEL staining. Athymic mice bearing IGROV1-CP xenografts were treated as described in (A).

In order to evaluate the effect of cisplatin-loaded nanoparticles on apoptosis, tumor tissue sections were stained with TUNEL for evaluating DNA fragmentation. Enumeration of apoptotic nuclei (about 200 cells were counted) was made on slides picked up at random by two independent experimenters. A cluster of apoptotic bodies were given as a single count, and apoptotic counts in different treatment groups were shown in Figure 7(A). Xenografts treated with cisplatin-loaded nanoparticles revealed higher apoptotic levels than the controls (saline and blank nanoparticles, *p*<0.05). Although total counts of apoptotic cells in tumors treated with cisplatin-loaded nanoparticles were slightly higher than those in tumors treated with conventional cisplatin, no significant difference in total counts of apoptotic cells in both treatment groups was observed, as shown in Figure 7(B) (*p*>0.05).

### Treatment efficacy *in vivo* studies

The treatment efficacy of cisplatin-loaded nanoparticles for IGROV1-CP cell xenograft in BALB/c nude mice was evaluated. After the average volume of the tumors was up to 100–130 mm^3^, the tumor-bearing mice were divided into four groups (n = 8) with minimal difference in weight and size of tumors among groups. In addition, the mice were treated with following regimens including saline, nanoparticles without cisplatin, free cisplatin, and cisplatin-loaded nanoparticles every four days. The dose of cisplatin-based treatments was 3 mg/kg body weight.

**Figure 8:**
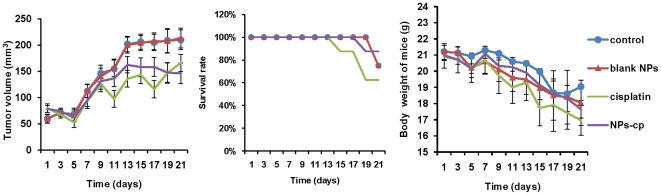
Effect of cisplatin-loaded nanoparticles on athymic mice. (A) Effect of free cisplatin (3 mg/kg) and cisplatin-loaded nanoparticles (NPs-cp, 3 mg/kg on the basis of cisplatin) on tumor growth in athymic mice bearing with IGROV1-CP cell xenografts. The mice were administered with prepared formulations at 4-day intervals in the total treatment period of 21 days. Saline solution was used as the control group, and the effect of blank nanoparticles (blank NPs) was also confirmed. The data were presented as Mean ± SD (n = 8 at the beginning of the experiment). (B) Survival rate of tumor-bearing athymic mice treated with cisplatin (3 mg/kg), NPs-cp (3 mg/kg on the basis of cisplatin), blank NPs and saline (control). (C) Body weight change as the treatment time of IGROV1-CP tumor-bearing mice. Bars indicated standard deviations.

Tumor size, body weight and survival rate of the mice were then monitored for 21 days following the beginning of treatment. The mean tumor volume at the end of the treatment period was 209±25 mm^3^ in saline-treated group, and 213±19 mm^3^ in blank nanoparticle-treated group, as shown in Figure 8(A). However, in cisplatin-treated group, the final mean tumor volume was 166±16 mm^3^, which was significantly smaller than that in saline-treated groups by ANOVA analysis at 95% confidence interval. Compared with the cisplatin-treated group, a slight reduction of tumor volume in cisplatin-loaded nanoparticles treated group with mean tumor size of 146±19 mm^3^ at end of treatment period. One possible reason was that the subsequent intracellular delivery of cisplatin may be hindered by mPEG modification of nanoparticles, which did not exhibit a significant contribution on tumor reduction. At the end of treatment period (days 21), the survival rates of the mice in the cisplatin-loaded nanoparticles-treated group, cisplatin-treated group, blank nanoparticle-treated group and saline-treated group were 87.5, 62.5, 75 and 75%, respectively, as shown in Figure 8(B). The difference in survival rates from four groups suggested that cisplatin-loaded nanoparticles were much safer than free cisplatin. The body weights of the mice exhibited a gradual decrease starting from the second dose although no significant difference in four groups was observed, as shown in Figure 8(C).

## Discussion

Physicochemical characteristics of cisplatin, such as poor solubility in water (1 mg/mL), high binding affinity to plasma proteins and degradability, may limit the therapeutic efficacy of cisplatin. Cisplatin-based obstacles on its clinical application can be resolved by using bioavailable polymeric nanoparticles. Currently, some types of nanoparticles prepared from poly(lactide)-monomethoxy-poly(ethyleneglycol) (PLA-mPEG) and PLGA-mPEG copolymers have gained increasing attention. The rationale of this study is to increase the loading and encapsulation efficiency of cisplatin by nanoparticles, thus improving the stability of cisplatin. TPGS also can provide a safe alternative of polyvinyl alcohol (PVA) or other additives for improving the preparation of uniform nanoparticles from batch to batch.

Using modified W/O/W double emulsion method developed by Gryparis EC et al. [20], we have developed a novel concept of “core-CMC-cisplatin-crosslinking” that can greatly enhance the loading efficiency of cisplatin. During the crosslinking of CMC, chloride ion in cisplatin can exchange hydrogen ions with CMC in water to form a complex. Due to the retention of the complex in the primary emulsion phase, cisplatin can be loaded into the nanoparticles made of hydrophobic PLGA with hydrophilic methoxy polyethylene glycol chains. The loading efficiency of cisplatin has improved to 3.9±0.3% (w/w), and the encapsulation efficiency is up to 70.9±2.6%. Furthermore, there are no reports related to the application of CMC polymers as a strategy to improve the loading efficiency of cisplatin. Therefore, cisplatin-loaded nanoparticles should be the effective drug delivery carriers for cisplatin *in vivo* study.

The high encapsulation efficiency of cisplatin is also partly due to the application of TPGS emulsifier in coordination-induced nanoparticle formation. TPGS is different from previously reported PVA, sodium cholate or other emulsifiers [17], [20]. TPGS, a water-soluble derivative of natural vitamin E, is commonly used in pharmaceutical and nutraceutical formulations. In this study, we have successfully generated homogeneous nanoparticles with the aid of TPGS. No debris and aggregation in the homogenous nanoparticles were observed by TEM. Monodisperse emulsion droplets favors coalescence without TPGS, and the nanoparticles are difficult to form. Here, TPGS is used with a hope to displace above mentioned emulsifiers and avoid their removal trouble.

During the evaluation of cell viability and treatment efficacy of prepared cisplatin-loaded nanoparticles, the viability of IGROV1-CP cells incubated with free cisplatin at the concentration range of 8–24 µM for three days exhibited a decrease from 79.6% to 14.8%, while the viability of the cells treated with cisplatin-encapsulated nanoparticles revealed a reduction from 76.9% to 10.8%. Moreover, the cisplatin-loaded nanoparticles effectively inhibited the tumor growth in nude mice when compared with free cisplatin at the same dose, suggesting that the nanoparticles did not interrupt anticancer effectiveness of cisplatin *in vivo*. TUNEL assay also confirmed the enhanced effectiveness of cisplatin-loaded nanoparticles on tumor inhibition. Meanwhile, histopathological examination revealed hepatic necrosis and atrophy in kidney in the group treated with free cisplatin, but these kinds of histopathological changes were not observed in the groups treated with cisplatin-loaded nanoparticles. In addition, other major organs such as heart, spleen and lung did not reveal abnormalities either. Thus, the core-CMC-cisplatin-crosslinked nanoparticles should be much safer and higher effectiveness than free cisplatin during cancer treatment.

In order to further understand the side effects of cisplatin-loaded nanoparticles, potential acute toxicity of cisplatin-loaded nanoparticles was also evaluated. ICR mice were intravenously injected with 0.8 mL of cisplatin-loaded nanoparticles at the cisplatin concentrations of 1.0, 1.5, 2.0, 2.5 and 3.0 mg/mL, respectively, and the survival rate of the mice was examined. As expected, the LD_50_ of cisplatin-loaded nanoparticles revealed 12-fold higher than that of free cisplatin. Comparing the overall survival rate, BALB/c mice showed more tolerance to cisplatin-loaded nanoparticles than free cisplatin, which indicated that cisplatin-loaded nanoparticles had more safety than free cisplatin at the condition of relatively high administration dose.

In general, cisplatin-loaded nanoparticles have been successfully designed and prepared. Spherical particles and narrow size distribution have been characterized. Sustained release of cisplatin over five days from PLGA-mPEG nanoparticles *in vitro* was observed. Therefore, we can conclude that nanoparticles have a significant contribution to the controlled release of cisplatin and do not damage the treatment effectiveness of cisplatin. Our findings provide further evaluation of cisplatin-loaded nanoparticles as a novel cancer therapy. However, in order to maximize the antitumor effect of cisplatin-loaded nanoparticles, it is necessary to further optimize the administration dose and injection regime.

Current strategies, especially for making such nanoparticles, involved multi-step preparation. As a result, the procedure is an inherently inefficient system, which may not be easily scalable and may result in batch-to-batch variation. In our laboratory, we have developed a new method for the preparation of nanoparticles with batch-to-batch uniformity. It is a simple, scalable, efficient and controllable system using our well-designed formulation strategy. Our proof-of-concept *in vitro* and *in vivo* evaluation shows that PLGA-mPEG nanoparticle formulation is a potential cisplatin delivery system for ovarian cancer treatment.

## Materials and Methods

### Materials

Cisplatin was purchased from Shandong Boyuan Chemical Co. Ltd. (Jinan, China). CMC was purchased from Aladdin Reagent Co. Ltd. (Shanghai, China). mPEG (M_W_ 5 kDa) and TPGS were obtained from Jiangsu XiXin Vitamin Co. Ltd. The reverse-phase column (ZORBAX-NH_2_, 250×4.6 mm, 5 µm) was purchased from Agilent Technologies Co. Ltd. (USA). All reagent water used in the laboratory was pretreated with the Milli-Q Plus System (Millipore Corporation, USA). All mPEG samples were dehydrated by azeotropic distillation with toluene, and then vacuum dried at 50°C for 12 h before use. IGROV1-CP cells were kindly provided by Dr. Stephen Collins at UCSD (CA, USA).

### Animals

All animal experiments were conducted with the approval of the Institutional Animal Ethics Committee (IACE) of the Research Center of Laboratory Animal Science of Zhejiang Chinese Medical University (Hangzhou, China). The permit numbers are Syxk (zhe) 2008-0115. ICR mice and BALB/c mice (4–6 weeks old and weighing 18–22 g) were kept at the animal center. Cisplatin-loaded nanoparticles were administered with intravenous injection. At the end of treatment periods, liver, kidney, heart, spleen and lung tissues were collected as per the approval of the IAEC.

### Synthesis and characterization of PLGA-mPEG copolymer

PLGA-mPEG copolymers were prepared by a melt polymerization process under vacuum using stannous-2-ethylhexanoate as catalyst [23]. The PLGA(30)-mPEG(5) was synthesized with composition LA∶GA∶EO = 3∶1∶1, Mw (the weight average molecular weight) = 3.7×10^4^, PI = 1.8 (LA, GA and EO stand for lactic acid, glycolic acid and ethylene oxide components respectively). The copolymer was characterized with ^1^H-NMR and GPC.

### Preparation and characterization of cisplatin-loaded nanoparticles

PLGA-mPEG nanoparticles loaded with cisplatin were prepared with W/O/W emulsion solvent evaporation method developed by Gryparis EC [20], with specific modifications. Briefly, 5.71 mg/mL cisplatin dissolved in 30 mM CMC aqueous solution was emulsified in an organic phase by using sonication (bioruptor, model UCD-200 TM-EX) at 100 W for 45 seconds. The organic phase was mainly composed of chloroform and acetone containing 50 mg PLGA-mPEG. Water-in-organic phase emulsion was added to an aqueous solution of TPGS (0.035%, w/v).

The resultant W/O/W emulsion was then microfludized with a minimum pressure of 12000 PSI, and agitated by a magnetic stirrer for 3 h at room temperature until complete evaporation of chloroform and acetone. Finally, the nanoparticle suspension was lyophilized and stored at 4°C until future use. In order to increase the entrapment of cisplatin in nanoparticles, CMC in the internal aqueous phase of W/O/W was conjugated to cisplatin in 1 mL of water for 3 hours with continuous gentle agitation. Blank nanoparticles were also prepared with the same method without cisplatin.

Particle size and zeta potential were measured by using Malvern Zetasizer instrument (13 runs per sample, NETASIZER NANO S90). The morphological examination of nanoparticles was observed under TEM (JEM-1230, JEOL, Japan). One drop of nanoparticle suspension was placed on a copper grid covered with nitrocellulose membrane and dried in the open air before negative staining with phosphotungstic sodium solution (1% w/v).

### Determination of loading efficiency and *in vitro* release rate of cisplatin

The loading efficiency of cisplatin in nanoparticles was determined by using a direct procedure [24]. Totally 5 mg of lyophilized nanoparticles was dissolved in 1 mL of NaOH (0.1 N) and agitated overnight on a magnetic stirrer at room temperature. The suspension was centrifuged at 18,000 g for 10 min, and 90 µL of supernatant was used to quantify cisplatin content by HPLC. The encapsulation efficiency was calculated as the amount of cisplatin recovered from nanoparticles relative to the initial amount of cisplatin used for each preparation. In addition, in order to quantify the release kinetics of cisplatin from nanoparticles, cisplatin-loaded nanoparticles (5 mg) were suspended in 10 mL of phosphate buffered saline (PBS). The suspension was placed into a microcentrifuge tube and then put in an orbital water bath shaking at 120 rpm at 37°C. The tubes were centrifuged at designated time intervals. After centrifugation, the supernatants were collected for the determination of cisplatin. After sampling our supernatants, the incubation medium was replaced by fresh PBS and the tubes were placed back in the incubator. The cisplatin content of these solutions was determined by measuring the absorbance at 310 nm with a non-gradient mobile phase consisting of 0.9% NaCl and methanol (v/v, 25/75) at a constant flow rate of 1.0 mL/min.

### *In vitro* cytotoxicity study

The MTT assay was used to evaluate the toxicity of blank nanoparticles, cisplatin-loaded nanoparticles and free cisplatin against IGROV1-CP ovarian cancer cells [25]. The cells were seeded into plastic 96-well plates at 3×10^3^ cells per well. Twenty-four hours after plating, cisplatin, blank nanoparticles and cisplatin-loaded nanoparticles (both suspended in culture medium) at various concentrations were added to the wells. A total of 50 µL of MTT solution (5 mg/mL in PBS, pH 7.4) was added into each well and incubated at 37°C for 3 hours. The solution was withdrawn, and then 200 µL of acidified isopropanol (0.33 mL HCl in 100 mL isopropanol) was added and agitated thoroughly to dissolve the formazan crystals. The solution was immediately read on a microplate reader (TECAN INFINIT M200, USA) at a wavelength of 490 nm. The experiments were performed independently three times. Cytotoxicity was expressed as inhibition percentage of cell viability.

### *In vivo* evaluation of acute toxicity

ICR mice were used to assess relative toxicity after intravenous administration of cisplatin-loaded nanoparticles. All mice were raised under specific pathogen-free (SPF) environments. All animal experiments were performed in full compliance with guidelines approved by the Animal Care Committee of Zhejiang Chinese Medical University. In order to determine median lethal dose (LD_50_), the mice were administered with 0.8 mL of cisplatin-loaded nanoparticles in saline solution at the doses of 40, 60, 80, 100 and 120 mg/kg through intravenous injection. Each group was 10 mice. The survival time of each animal was recorded up to 14 days and LD_50_ was calculated according to the Bliss mathematical scheme.

### HE staining

After treatment with five consecutive different formulations, BALB/c mice were sacrificed by overdose of ether inhalation anesthesia. Macroscopic examination and histological analysis of heart, lung, liver, spleen and kidney tissues were performed. The organs were firstly fixed in 10% neutral-buffered formalin solution, and then embedded in paraffin. Embedded sections were cut at 5 µm and stained with hematoxylin and eosin (HE). The images were acquired at 400× magnification. Detail histopathological and morphological changes were reviewed by a pathologist at Zhejiang University of Traditional Chinese Medicine.

### In situ cell death detection of ovarian cancer xenografts

The tumors in cisplatin-loaded nanoparticles (3 mg/kg once per 4 days) treated mice were sequentially excised, formalin-fixed and paraffin-embedded. Embedded tissue samples were examined by TUNEL assay as described previously [25]. Briefly, tissue sections were dewaxed and rehydrated according to standard protocols (*e.g.*, by heating at 60°C followed by washing in xylene and rehydration through a graded series of ethanol and double distilled water). After two more washes in PBS, the slides were incubated with proteinase K solution for 30 minutes at room temperature. After rinsing slide(s) twice with PBS, reaction mixture (consisting of Enzyme Solution and Label Solution) in the TUNEL detection kit was then added and incubated at 37°C in the dark for 1 hour. Slides were rinsed with PBS for 3 times, and the samples were directly analyzed under an OLYMPUS IX-71 microscope. An excitation wavelength in the range of 488 nm and emission wavelength in the range of 520 nm (green) was used. Apoptotic cells were photographed with microscope.

### *In vivo* antitumor efficacy

The antitumor efficacy of cisplatin-loaded nanoparticles was evaluated by using BALB/c female mice bearing IGROV1-CP cells. Cell suspension (7×10^6^ per mice) was prepared and inoculated into the subcutaneous dorsa of mice. Nearly two weeks after inoculation (when mean tumor volume was approximately 100–130 mm^3^), tumor-bearing mice (eight mice per group) were injected with saline, blank nanoparticles, free cisplatin and cisplatin-loaded nanoparticles via the lateral tail vein. The administrations were given once every 4 days at the cisplatin dose of 3 mg/kg. The treatment period was 3 weeks. The tumor volume was calculated by using a×b^2^/2, where a is the largest diameter and b is the smallest diameter. In addition, the toxicities of free cisplatin and cisplatin-loaded nanoparticles were evaluated by monitoring the changes in body weight and survival rates.

### Statistical analysis

The difference in tumor volume, survival rate and tumor weight between the treatment and control groups was assessed by analysis of variance. The data were normally distributed. All data were presented as Mean ± SD. All statistical analysis was carried out by SPSS Version 11.0 software. A significant difference was considered at *p*<0.05.
